# Depletion of RIPK4 parallels higher malignancy potential in cutaneous squamous cell carcinoma

**DOI:** 10.7717/peerj.12932

**Published:** 2022-02-10

**Authors:** Jing Xu, Dongping Wu, Bicheng Zhang, Chi Pan, Yinglu Guo, Qichun Wei

**Affiliations:** 1 Department of Radiation Oncology, The Second Affiliated Hospital and Cancer Institute (National Ministry of Education Key Laboratory of Cancer Prevention and Intervention), Zhejiang University School of Medicine, Hangzhou, China; 2 Department of Radiation Oncology, Shaoxing People’s Hospital, Shaoxing Hospital of Zhejiang University, Shaoxing, China; 3 Department of Breast Surgey, The Second Affiliated Hospital, Zhejiang University, College of Medicine, Hangzhou, China

**Keywords:** RIPK4, Cutaneous squamous cell carcinoma, Radiosensitivity, Signaling pathways, Therapeutic target

## Abstract

**Background:**

The RIPK4 (receptor-interacting protein kinase 4), a member of the RIPK family, acts as an important regulator of epidermal differentiation, cutaneous inflammation, and cutaneous wound repair. However, Until now, the role of RIPK4 in tumorigenesis remains elusive. There have been no studies exploring the effects of RIPK4 on the signaling pathway in cutaneous squamous cell carcinoma (SCC). It remains unknown whether RIPK4 expression, which can affect the degree of epidermal differentiation can also influence the radiosensitivity of skin SCC. It is urgent to fully elucidate the biological mechanism by which RIPK4 promotes carcinogenesis in skin SCC and determine whether RIPK4 expression levels predicts the sensitivity to radiotherapy in skin SCC.

**Methods:**

Human skin SCC cell line, A431, was transfected with either small interfering RNAs (siRNAs) targeting RIPK4 (siR-RIPK4) or negative control siRNA (siR-NC). Western blotting was used to detect the expression of RIPK4 and Raf/MEK/ERK pathway-related proteins. The cells were irradiated using an X-ray irradiator at 6 MV with different radiation doses (0, 2, 6, and 10 Gy). Cell proliferation analysis, colony formation assay, transwell cell migration and invasion assay, cell cycle and apoptosis analysis were conducted to investigate the effect of RIPK4 silencing on skin SCC malignancy and radiosensitivity.

**Results:**

RIPK4 protein expression was significantly decreased in the A431 cells transfected with siR-RIPK4, compared with the A431 cells transfected with siR-NC. RIPK4 silencing facilitated the proliferation, colony formation, migration, and invasion ability of A431 cell line, while cell cycle progression or cell apoptosis were not significantly influenced. In contrast with the previous literature, Raf/MEK/ERK pathway was not effected by RIPK4 knockdown in skin SCC. RIPK4 knockdown could not reverse the radiation resistance of A431 cells to irradiation *in vitro*.

**Conclusions:**

In general, although depletion of RIPK4 cannot reverse the radiation resistance of A431 cells *in vitro*, it parallels higher malignancy potential in cutaneous SCC. To our knowledge, this is the first report of the effects of RIPK4 expression on the Raf/MEK/ERK signaling pathway and radiosensitivity in cutaneous SCC. The better understanding of the molecular mechanism of RIPK4 in cutaneous SCC may provide a promising biomarker for skin SCC prognosis and treatment.

## Introduction

RIPK4 (receptor-interacting protein kinase 4) belonged to the RIPK family, and it was initially identified interacting with protein kinase C (PKC) β and PKCδ in yeast two-hybrid assays (Bahr et al., 2000; Chen et al., 2001). RIPK4 contains an N-terminal RIP-like kinase domain, a unique intermediate domain that can be cleaved by caspases, and a C-terminal region characterized by the presence of 11 ankyrin repeats (Meylan et al., 2002). Distinct from other RIPK family members, RIPK4 plays a pivotal role in epidermal differentiation, cutaneous inflammation, and cutaneous wound repair (Rountree et al., 2010; Adams & Munz, 2010; Adams et al., 2007). RIPK4-deficient animals are characterized by abnormal epidermal differentiation and RIPK4 knockout in mice results in perinatal lethality, which is most likely due to the suffocation caused by dysplastic epidermis (Holland et al., 2002). In humans, homozygous mutation of RIPK4 have been linked to a lethal autosomal-recessive disorder called Bartsocas-Papas syndrome (BPS), which is typically characterized by loss of epidermal differentiation and exhibits severe ectoderm-originated organ anomalies as seen in mice (Mitchell et al., 2012; Kalay et al., 2012).

To date, the critical role of RIPK4 in tumorigenesis has not been extensively investigated. By large-scale genomic sequencing analysis, RIPK4 mutations have been identified in cutaneous squamous cell carcinoma (SCC), esophageal SCC, human papillomavirus (HPV)-positive oral SCC, and head and neck SCC, implicating a critical function of RIPK4 in squamous differentiation and carcinogenesis (Stransky et al., 2011; Li et al., 2018; Gillison et al., 2019; Pickering et al., 2014; Li et al., 2015). In addition, RIPK4 plays a conflicting role as either a tumor suppressor or a tumor promoter in different tumor types. Hence, RIPK4 may have a context-specific function in different cancer progression and signal transduction (Xu, Wei & He, 2020). RIPK4 expression is up-regulated and relates with a poor prognosis in ovarian cancer, cervical SCC, pancreatic cancer, bladder cancer and osteosarcoma (Qi et al., 2018; Liu et al., 2015; Liu et al., 2018; Yi et al., 2020; Liu et al., 2021). However, the expression of RIPK4 is down-regulated and positively correlates with favorable prognosis in tongue SCC, hepatocellular carcinoma (HCC) and lung adenocarcinoma (Wang et al., 2014; Kopparam et al., 2017; Li et al., 2021).

The classical Ras/Raf and MAPK pathway plays a critical role in epidermal homeostasis and carcinogenesis (Kern, Niault & Baccarini, 2011; Khavari & Rinn, 2007). In epidermal keratinocytes, RIPK4 could phosphorylate the N-terminal domain of Pkp1 (plakophilin-1) and enhance epidermal differentiation by functionally suppressing Ras/MAP kinase signaling pathway (Lee et al., 2017). In contrast, RIPK4 facilitated pancreatic cancer cell migration and invasion *via* the phosphatidylethanolamine binding protein 1 (PEBP1) degradation-induced activation of the RAF1/MEK/ERK pathway (Qi et al., 2018). Until now, there have been no studies exploring the effects of RIPK4 on the Raf/MEK/ERK signaling pathway in cutaneous SCC cells. Meanwhile, RIPK4 is essential for epidermal differentiation, and during cancer progression, poorly differentiated cancers appear to present a higher degree of cell migration, thus promoting the dissemination of cells from the tumor mass (Thomas & Speight, 2001). Tumor-specific characteristics, such as differentiation status, is related to intrinsic radiosensitivity of tumor cells, and well differentiated tumors are generally less sensitive to radiation than the anaplastic tumors (Vogin & Foray, 2013). It remains unknown whether RIPK4 expression can influence the radiosensitivity of skin SCC. It is urgent to fully elucidate the biological mechanism by which RIPK4 promotes carcinogenesis in skin SCC, and whether RIPK4 expression level predicts the sensitivity to radiotherapy in skin SCC.

## Materials and Methods

### Cell culture and transfection

Skin SCC cell line A431 was obtained from Procell Biological Technology (Wuhan, China). The A431 cell line was cultured in DMEM (Gibco; Thermo Fisher Scientific, Inc., Waltham, MA, USA) supplemented with 10% fetal bovine serum (FBS) and antibiotics (1% penicillin/streptomycin) and incubated at 37 °C in 5% CO_2_.

Small interfering RNAs (siRNAs) targeting RIPK4 (siR-RIPK4) and negative control siRNA (siR-NC) were synthesized by Shanghai GenePharma Pharmaceutical Technology Co., Ltd. (Shanghai, China). The siR-RIPK4 and siR-NC sequences were as follows: siR-RIPK4 #1, 5′-CCACUACCACGUCAAGAUUTT-3′ (sense), 5′-AAUCUUGACGUGGUAGUGGGC-3′ (antisense); siR-RIPK4 #2, 5′-GCACGAUGUAUACAGCUUUTT-3′ (sense), 5′-AAAGCUGUAUACAUCGUGCTT-3′ (antisense); siR-NC, 5′-UUCUCCGAACGAGUCACGUTT-3′ (sense), 5′-ACGUGACUCGUUCGGAGAATT-3′ (antisense). Cells were transfected with either siR-RIPK4 or control siR-NC using the lipofectamine 2000 (Invitrogen, Carlsbad, CA, USA) according to the instructions of manufacturer. The cells were infected 24 h later when they had reached 70%–80% confluence. The protein levels were detected 24 h after transfection.

### *In vitro* radiation therapy

The irradiation exposure was performed using an X-ray irradiator (Rad Source Technologies, RS2000-PRO, Burford, GA, USA). The cells were irradiated by X-ray at 6 MV with different radiation doses (0, 2, 6, and 10 Gy).

### Cell proliferation analysis

First, 2 × 10^3^ cells per well were seeded into the 96-well plate. Then, 10 μl Cell Counting Kit-8 (CCK8; Dojindo Molecular Technologies, Inc., Shanghai, China) was added to each well 24, 48, 72, and 96 h after incubation. The absorbance was determined using the Thermo Scientific Microplate Reader (Thermo Fisher Scientific, Waltham, MA, USA) at 450 nm. The cell proliferation curve was generated according to the results above.

### Transwell cell migration and invasion assay

The cell migration and invasion assays were both performed using a 24-well Transwell chamber with a pore size of 8 μm (Corning, Lowell, MA, USA). For the invasion assay, the inserts were previously coated with 100 μL of extracellular matrix gel (BD Biosciences, San Jose, CA, USA; 1:40 diluted in DMEM). Cells transfected with siR-RIPK4 or siR-NC were seeded on the upper chambers containing 300 μL of serum-free DMEM medium, and the chambers were then placed in the 24-well plate with medium contained 10% FBS as a chemoattractant. After incubation for 24 h at 37 °C with 5% CO_2_, the nonmigrated or noninvaded cells in the upper chambers were removed with cotton swabs, and the cells on the lower surface of the filter were fixed with 4% paraformaldehyde for 15 min and then stained with 0.05% crystal violet for another 15 min. The number of the migrated or invaded cells was counted by five random visual fields at ×40 magnification under a microscopy. The experiments were carried out in triplicates.

### Cell cycle and apoptosis analysis by flow cytometry

The A431 cells transfected with siR-RIPK4 and siR-NC were harvested 24 h after transfection. Cells were then collected by trypsinization, washed with ice-cold phosphate-buffered (PBS) and fixed in 70% ethanol overnight. The cells were pretreated with RNase (50 μg/mL) for 30 min at 37 °C and incubated with 50 μg/mL propidium iodide (PI) for 30 min under dark conditions. Cell cycle distribution was evaluated by using flow cytometer (BD Bioscience).

For the cell apoptosis assay, cells were digested with 0.25% trypsin (without EDTA), washed in 4 °C pre‑cooled PBS and were resuspended in 1× Binding Buffer. A total of 100 µL cell suspension was stained with 5 µL Annexin V-fluorescein isothiocyanate (Annexin V-FITC) (Apoptosis Detection kit, 4A Biotech Co. Ltd., Beijing, China) for 5 min at room temperature in the dark. Subsequently, cells were stained with 10 µl PI and assessed using a flow cytometer and results were analyzed using FlowJo software (Tree Star, Ashland, OR, USA). The assay was performed in triplicate.

### Colony formation assay

The A431 Cells were transfected with siR-RIPK4 and siR-NC for 24 h and plated in 6-well plates containing 0.5 × 10^3^ cells per well in triplicate. After 14 days, colonies were washed with PBS and fixed with 10% methanol. Fixed colonies were stained with crystal violet, and colonies containing more than 50 cells were counted.

### Western blot analysis

The expression of RIPK4 and Raf/MEK/ERK pathway-related proteins in A431 cells transfected with siR-RIPK4 or siR-NC were analyzed by Western blot. Cells were harvested after being washed with PBS twice, lysed with RIPA buffer, and centrifuged at 14,000 rpm for 7 min at 4 °C. The protein concentrations were measured using the Pierce BCA Protein Assay kit (Thermo Fisher Scientific Inc., Waltham, MA, USA). Equal amounts of protein (20 µg/load) from each sample were loaded on sodium dodecyl sulfate polyacrylamide gel electrophoresis (SDS-PAGE) electrophoresis and transferred to polyvinylidene fluoride (PVDF) membranes and reacted with primary antibodies overnight at 4 °C. Following incubation with the secondary antibodies for 2 h at room temperature, the protein bands were detected using the enhanced chemiluminescence (ECL kit; Bio-Rad, Hercules, CA, USA) following the manufacturer’s instructions.

Antibodies against the following proteins were obtained from Cell Signaling Technology (Danvers, MA, USA): p-MEK (#9154S), p-ERK (#4370), Raf (#9422), p-Raf (#9427), ERK (#4695), and Myc (#5605). Antibody to MEK (#ab178876) and p-Myc (#ab185656) were obtained from Abcam (Cambridge, MA, USA). Antibody against RIPK4 (#A8495) was obtained from ABclonal (Wuhan, China). All these antibodies mentioned above were diluted at 1:1,000. β-actin (A5441; Sigma-Aldrich, St. Louis, MO, USA) was used as an internal control. Anti-Rabbit IgG antibody (#7074) and Anti-Mouse IgG antibody (#7076) which were purchased from Cell Signaling Technology were used as the secondary antibodies at a dilution of 1:5,000.

### Statistical analysis

The results were expressed as the means ± SE. For analysis, two-tailed unpaired Student’s t-tests, one-way analysis of variance, or Pearson’s χ2 tests were used to evaluate the data. All statistical analyses were performed using SPSS 22.0 software (IBM Corp., Armonk, NY, USA). *P* < 0.05 was considered to be statistically significant. *P < 0.05; **P < 0.01.

## Results

### RIPK4 silencing promoted the proliferation, colony formation, migration, and invasion ability of A431 cell line

**Figure 1:**
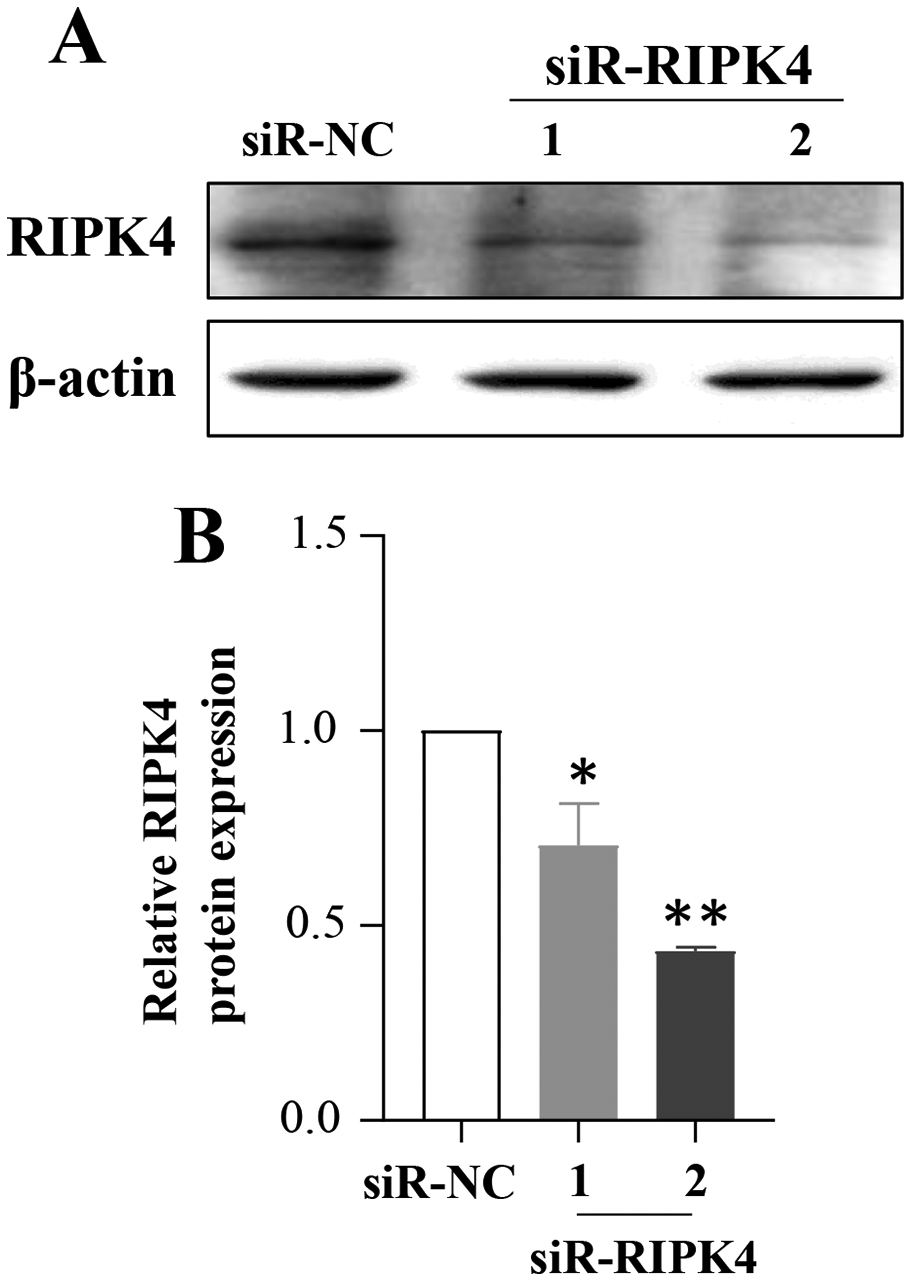
A431 cell line was transfected with RIPK4 siRNA and negative control siRNA. (A) The expression level of RIPK4 in A431 cells transfected with RIPK4 siRNA and negative control siRNA was assessed by western blotting. β-actin was used as the loading control. (B) ImageJ densitometric analysis of the relative expression of RIPK4. Error bars represent mean ±S.D. of three independent experiments, each conducted in triplicate. *P< 0.05, **P < 0.01 *vs* control group. RIPK4, receptor interacting protein kinase 4; siRNA, small interfering RNA; NC, negative control.

To determine whether *in vitro* RIPK4 plays an important role in regulating cell growth, colony formation and affects the cellular migration and invasion, the A431 cell line was transfected with siR-NC and siR-RIPK4. Two independent siRNA sequences were used to knock down the RIPK4 expression in A431 cells. RIPK4 expression was examined by western blot. By western blot, RIPK4 protein expression was significantly decreased in the A431 cells transfected with siR-RIPK4-1 and siR-RIPK4-2, compared with the A431 cells transfected with siR-NC (Fig. 1). siR-RIPK4-1 and siR-RIPK4-2 generated the consistent knockdown results in A431 cell line and were thus chosen for further studies.

**Figure 2:**
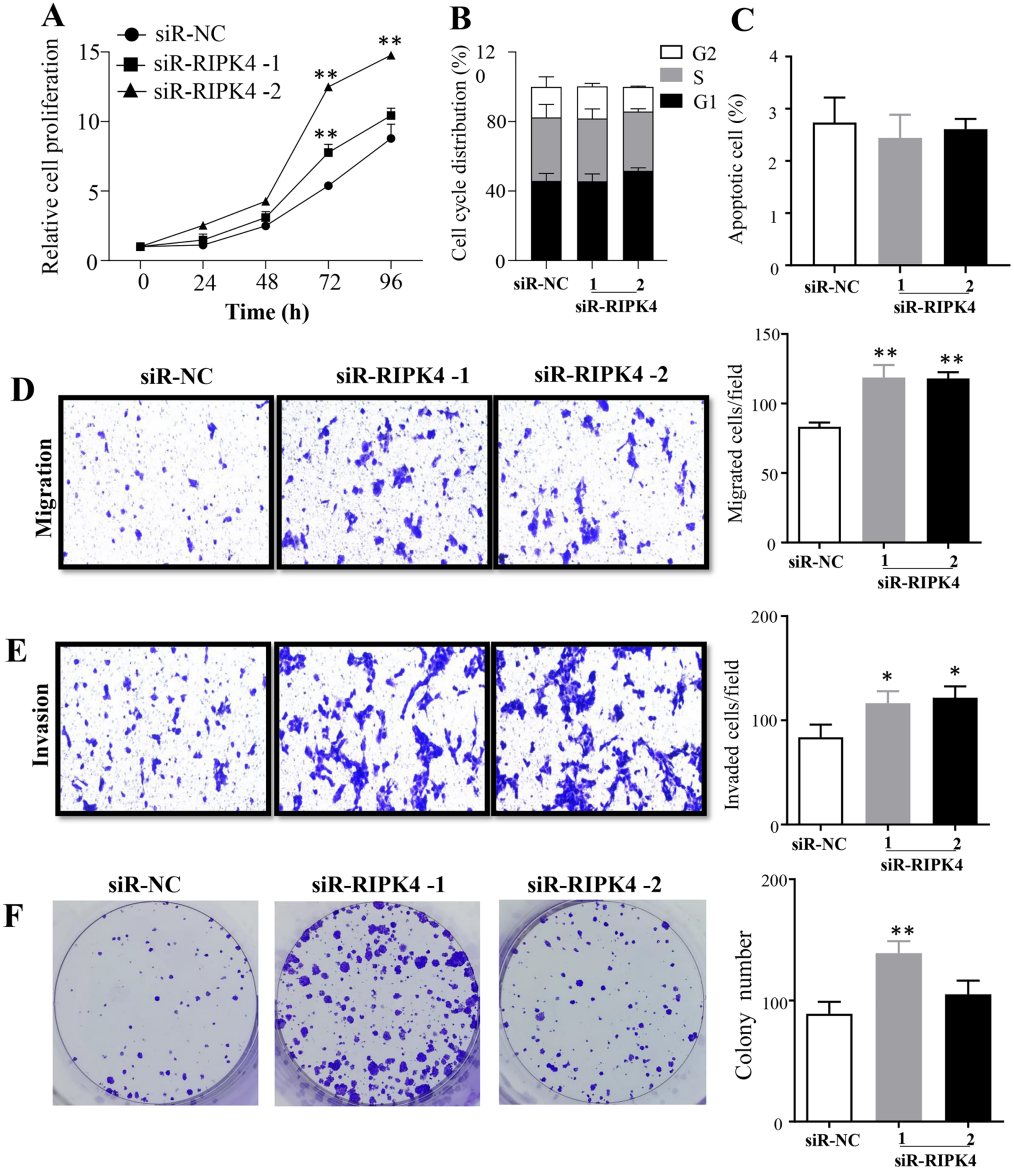
RIPK4 silencing promoted cell proliferation, migration and invasion in A431 cells. (A) Cell proliferation of A431 cells after silencing RIPK4 was measured by Cell Counting Kit-8 assay. (B) Cell cycle analysis of A431 cells transfected with RIPK4 siRNA and negative control siRNA were examined by flow cytometry. (C) A431 cells transfected with RIPK4 siRNA and negative control siRNA were stained with Annexin V-FITC/PI and analyzed by flow cytometry. (D) Cell migration and (E) invasion of A431 cells after RIPK4 silencing were measured by migration and invasion assays (magnification, ×40). (F) Colony counts in A431 cells transfected with RIPK4 siRNA and negative control siRNA. Error bars represent mean ±S.D. of three independent experiments, each conducted in triplicate. *P < 0.05, **P < 0.01 *versus* control group. RIPK4, receptor interacting protein kinase 4; siRNA, small interfering RNA; NC, negative control.

The effects of RIPK4 on the growth and colony formation of A431 cells were assessed using MTT assay and colony formation assay, respectively. Down-regulation of RIPK4 enhanced the proliferation and colony formation ability of A431 cells (Figs. 2A, 2F). Furthermore, correlating with the decline of RIPK4 expression in cells transfected with siR-RIPK4, significant increases in migration and invasiveness were observed in A431 cells (Figs. 2D, 2E). In contrast, knocking down RIPK4 in A431 cell line had no obvious effect on cell cycle progression or apoptosis when measured by flow cytometry (Figs. 2B, 2C). Collectively, these data suggested that RIPK4 played an important role in the growth, colony formation, migration, and invasiveness ability of A431 cells.

### RIPK4 knockdown could not reverse the radiation resistance of A431 cells to irradiation

**Figure 3:**
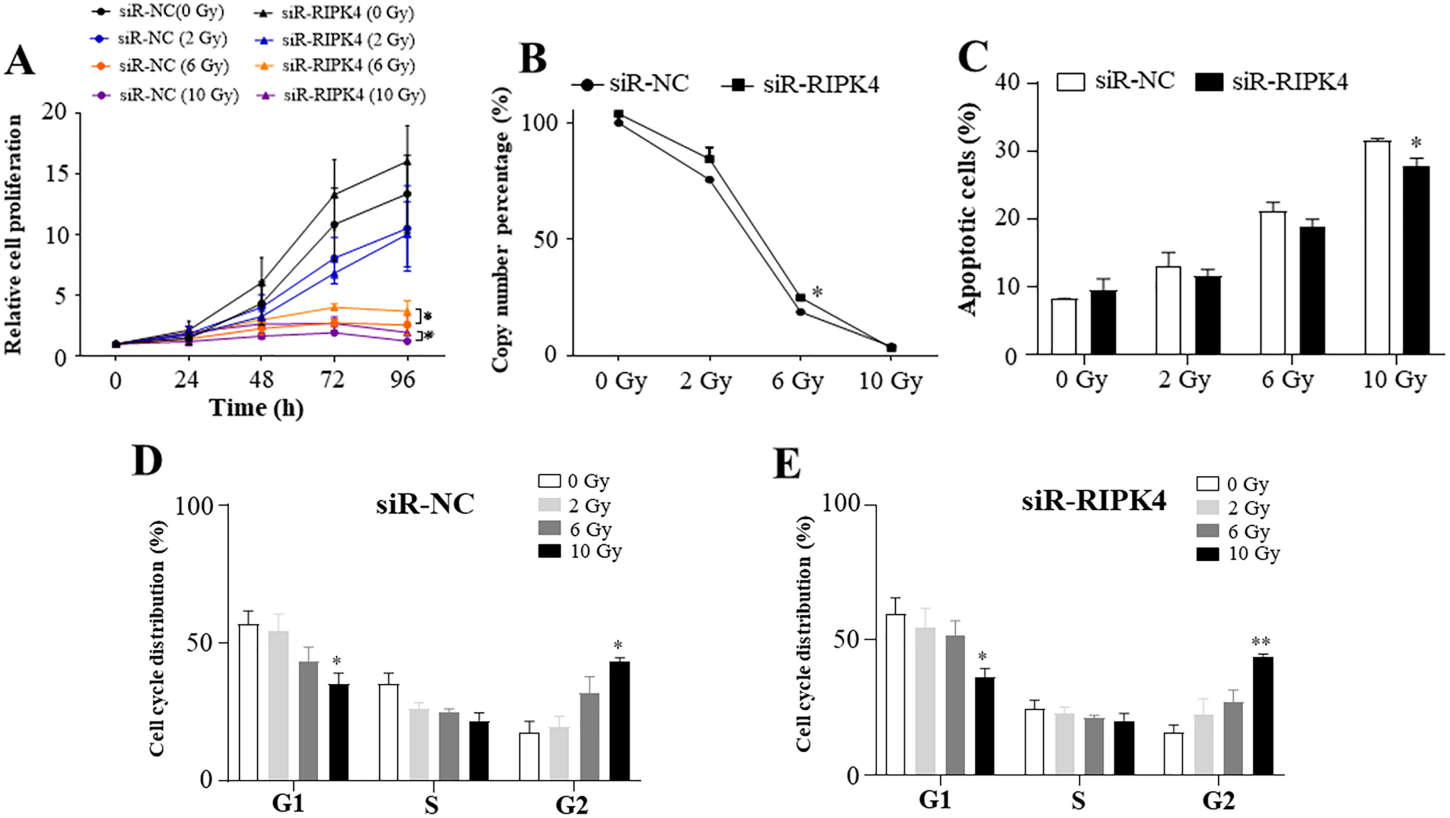
RIPK4 knockdown could not reverse the radiation resistance of A431 cells to irradiation. A431 cells were transfected with RIPK4 siRNA and negative control siRNA. (A) Cell proliferation of A431 cells irradiating of different radiation doses was measured by Cell Counting Kit-8 assay. (B) Colony counts in A431 cells irradiating of different radiation doses were analysed. (C) A431 cells irradiating of different radiation doses were stained with Annexin V-FITC/PI and analyzed by flow cytometry. (D), (E) Cell cycle analysis of A431 cells irradiating of different radiation doses were examined by flow cytometry. Error bars represent mean ±S.D. of three independent experiments, each conducted in triplicate. *P < 0.05, **P < 0.01 *versus* control group. RIPK4, receptor interacting protein kinase 4; siRNA, small interfering RNA; NC, negative control.

To assess the effects of RIPK4 expression on radiosensitivity of A431 cells following irradiation, siR-RIPK4-1 was chosen for the further analysis. Our results indicated that irradiation inhibited A431 cell proliferation in a radiation dose-dependent manner, while knockdown of RIPK4 expression could not reverse radiation resistance of the irradiated A431 cells. RIPK4 silencing further significantly increased the attenuated proliferation in the A431 cells after exposure to the radiation doses of 6 Gy and 10 Gy (Fig. 3A). Our results also showed that irradiation dramatically suppressed colony formation ability of A431 cell line, and at the radiation dose of 6 Gy, colony formation ability of the A431 cells transfected with siR-RIPK4 was stronger than A431 cells transfected with siR-NC (Fig. 3B). It was also demonstrated that irradiation facilitated A431 cell apoptosis, while knockdown of RIPK4 further weakened the apoptosis mediated by ionizing radiation in the A431 cells at the radiation dose of 10 Gy (Fig. 3C). Radiation-induced G2 arrest was observed at a relatively high radiation dose of 10 Gy, otherwise, there were no significant differences of the cell cycle regulation after exposure to ionizing radiation between the A431 cells transfected with siR-NC and siR-RIPK4. Collectively, the loss of RIPK4 expression using siRNA could not reverse the radioresistance of A431 cells to various doses of ionizing radiation.

### Raf/MEK/ERK pathway was not activated by RIPK4 knockdown

**Figure 4:**
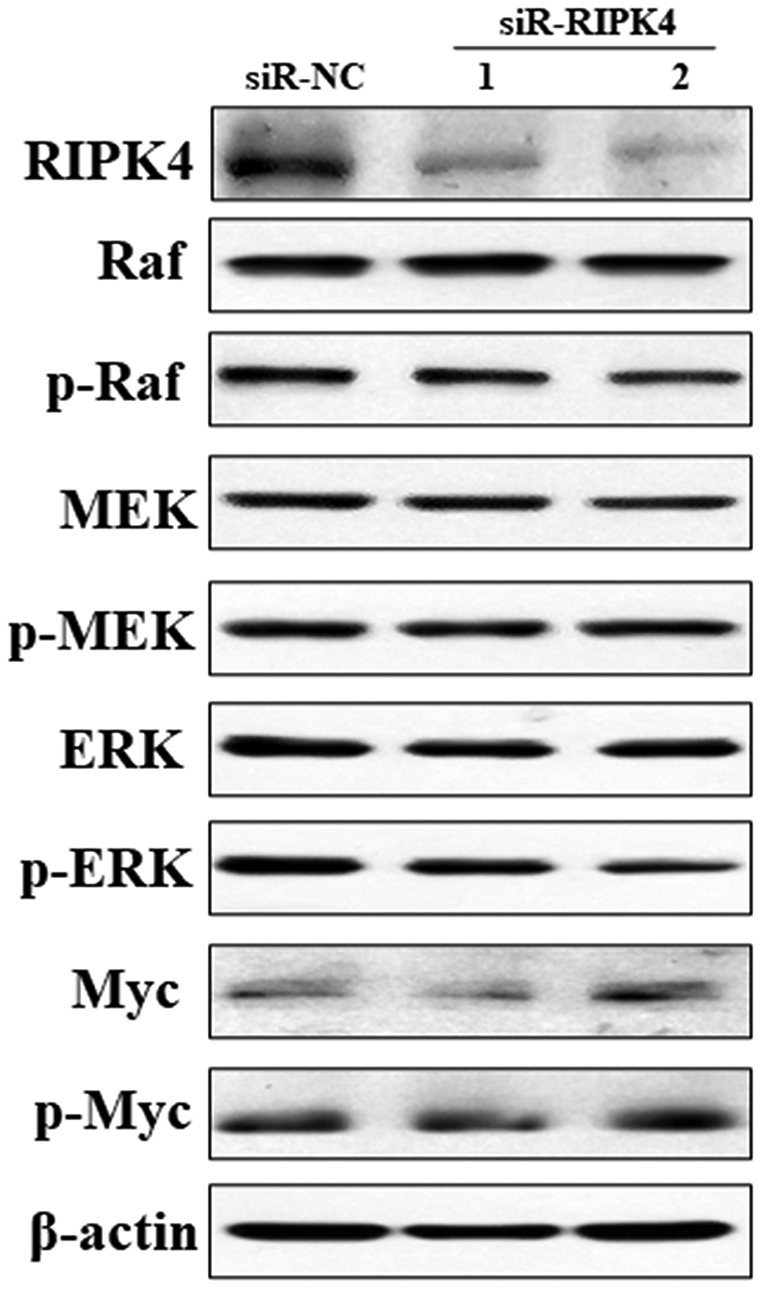
Silencing of RIPK4 did not affect Raf/MEK/ERK pathway. A431 cells were transfected with RIPK4 siRNA and negative control siRNA. RIPK4, Raf, p-Raf, MEK, p-MEK, ERK, p-ERK, Myc and p-Myc in A431 cells were assessed by western blot. β-actin was used as the loading control. RIPK4, receptor interacting protein kinase 4; siRNA, small interfering RNA; NC, negative control.

To decipher the molecular mechanism of RIPK4 in promoting the proliferation, colony formation, migration, and invasion of A431 cells, Raf/MEK/ERK pathway was measured in siR-RIPK4 and siR-NC-transfected cells. The results of western blot analysis showed that phosphorylation of Raf, MEK, ERK, and Myc expression levels were not significantly affected after RIPK4 silencing in A431 cells when compared with control siRNA (Fig. 4). These data thus indicated that RIPK4 might not be involved in the Raf/MEK/ERK signaling activation in skin SCC.

## Discussion

Collectively, our study demonstrated that depletion of RIPK4 in skin SCC cell line using siRNA promoted cell proliferation, colony formation, migration and invasion capacity. Hence, RIPK4 might act as a novel tumor suppressor gene in skin SCC. Furthermore, although RIPK4 was related with epidermal differentiation, RIPK4 knockdown could not reverse the relative radiation resistance of A431 cells to irradiation. In contrast with the previous studies, RIPK4 knockdown was not associated with the activation of Raf/MEK/ERK signaling pathway. Further studies were needed to explicit the molecular mechanism involved in skin SCC upon RIPK4 depletion.

Understanding the upstream pathways that regulate RIPK4 provides valuable information about how RIPK4 functions at the biochemical level and how it influences keratinocyte differentiation and carcinogenesis (Xu, Wei & He, 2020). However, so far, little is known regarding the activation mechanisms of RIPK4. Upon PKC activation, RIPK4 is hyper-phosphorylated and can be degraded by the SCF^β-TrCP^-mediated proteasomal degradation pathway to maintain cortical actin organization in keratinocytes (Tanghe et al., 2018). RIPK4 is also identified as a target gene of miR-330-3p, which can suppress the proliferation, migration and invasion of ovarian cancer cells (Cai et al., 2021).

In addition, RIPK4 may be involved in various carcinogenic molecular mechanisms in different types of tumors, including the nuclear factor-κB (NF-κB), Wnt/β-catenin pathway, and RAF/MEK/ERK pathway. NF-κB pathway can be activated by RIPK4 in both a kinase-dependent and kinase-independent manner (Moran et al., 2003). RIPK4 regulates constitutive NF-κB activity as well as NF-κB activation induced by B cell–activating factor of tumor necrosis factor family (BAFF) in diffuse large B-cell lymphoma (DLBCL) cells. Suppression of RIPK4 expression impairs the survival of DLBCL cells *in vitro* and inhibits tumor growth of xenografted DLBCL cells in mice (Kim et al., 2008). In nasopharyngeal carcinoma, RIPK4 is found to enhance the interaction between IKKβ and IKKα and activate NF-kB signaling pathway (Gong et al., 2018). Phosphorylation of DVL2, which is a receptor protein of the Wnt pathway, at Ser^298^ and Ser^480^ by RIPK4 favors canonical Wnt/β-catenin signaling (Huang et al., 2013). RIPK4 overexpression contributes to the progression of ovarian cancer in a xenograft tumor model. RIPK4 is also significantly upregulated in osteosarcoma and RIPK4 knockdown suppress epithelial to mesenchymal transition (EMT) by inactivating Wnt signaling (Yi et al., 2020). However, RIPK4 may only play an oncogenic role in Wnt-dependent tumors, since RIPK4 knockdown do not affect Wnt3a-induced β-catenin accumulation in pancreatic, kidney, and breast cancer cells (Huang et al., 2013). In pancreatic cancer, RIPK4 can promote cell migration and invasion *via* the phosphatidylethanolamine binding protein 1 (PEBP1) degradation-induce activation of the RAF1/MEK/ERK signaling pathway (Qi et al., 2018). In contrast, RIPK4 can phosphorylate the N-terminal domain of Pkp1 and suppress Ras/MAP kinase signaling pathway in epidermal keratinocytes (Lee et al., 2017). However, in our present study, RIPK4 knockdown did not affect Raf/MEK/ERK pathway in skin SCC cell line. Hence, the context-specific function of RIPK4 in signal transduction mechanisms may depend on the different tumor types. Furthermore, Liu et al utilized natural halloysite nanotube (HNT)–assisted delivery of an active siRNA targeting RIPK4 significantly inhibited RIPK4 expression and suppressed bladder cancer progression, with no adverse effects (Liu et al., 2019). In future, novel RIPK4-interacting pathways and precise therapeutic targets need to be further identified in order to elucidate the mechanisms underlying squamous epidermal carcinogenesis.

**Figure 5:**
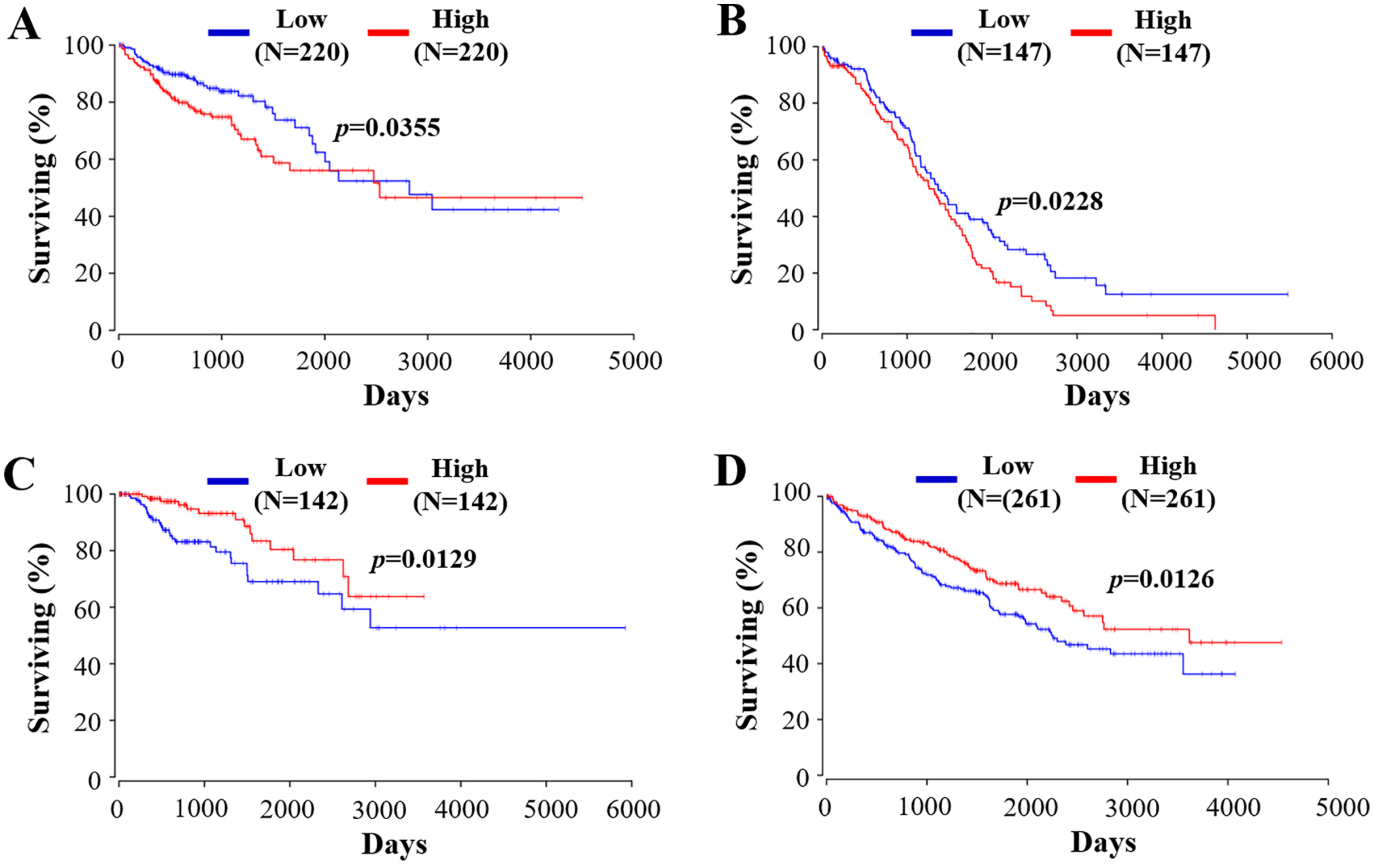
The prognostic value of RIPK4 in different types of tumor. Prognostic values of RIPK4 in (A) colon adenocarcinoma, (B) ovarian serous cystadenocarcinoma, (C) kidney renal papillary cell carcinoma, and (D) kidney renal clear cell carcinoma were obtained in Oncolnc. RIPK4, receptor interacting protein kinase 4.

RIPK4 plays a crucial role in the evaluation of prognostic in a variety of tumors. As stated above, RIPK4 expression is confirmed to be positively associated with favorable prognosis in tongue SCC, lung adenocarcinoma, and hepatocellular carcinoma (Wang et al., 2014; Kopparam et al., 2017; Li et al., 2021). By contrast, RIPK4 overexpression predicts poor prognosis in patients with ovarian cancer, cervical SCC, pancreatic cancer, bladder cancer, and osteosarcoma (Qi et al., 2018; Liu et al., 2015; Liu et al., 2018; Yi et al., 2020; Liu et al., 2021). We then used public data set OncoLnc (http://www.oncolnc.org) (Anaya, 2016) to perform survival analyses of other tumor types. RIPK4 can function as an independent prognostic indicator of worse outcome in colon adenocarcinoma (COAD) and in ovarian serous cystadenocarcinoma (OV) while RIPK4 overexpression predicts better outcome in kidney renal papillary cell carcinoma (KIRP) and kidney renal clear cell carcinoma (KIRC) (Fig. 5). However, the potential role of RIPK4 in the prognosis of skin SCC still remains unknown. Our study demonstrated that RIPK4 knockdown promoted cell proliferation, colony formation, migration and invasion capacity in skin SCC cell line. Consistent with our findings, RIPK4 might be involved in the process of bone metastasis from breast cancer. RIPK4 overexpression significantly reduced migration and invasion capacity of human breast cancer cell line MCF7 (Zhang, He & Zhang, 2019). Further studies were needed to clarify the role of RIPK4 in the prognosis of skin SCC and other different kinds of cancers.

It was in accordance with our study that no matter the RIPK4 expression level was upregulated or downregulated, there were no significant changes of apoptosis or cell cycle progression in human tongue SCC cell line Tca-8113 (Wang et al., 2014). The effect of RIPK4 in apoptosis and cell cycle arrest in other types of cancer cell lines should further be explored.

RIPK4 is identified as a key regulator of keratinocyte differentiation and RIPK4 deficiency in the skin epidermis greatly impairs skin differentiation (Xu, Wei & He, 2020). Bergonié and Tribondeau proposed what has become known as the basic law of radiobiology, stating that the tumor cell radiosensitivity is inversely proportional to its degree of differentiation (Vogin & Foray, 2013). Hence, we next examined the relationship between radiosensitivity of A431 cells and RIPK4 expression level. However, in the present study, decrease of RIPK4 in A431 cells could not reverse the radiation resistance at the dose of 2, 6, and 10 Gy, and it might be partly explained by the fact that RIPK4 knockdown promoted cell proliferation, colony formation, migration and invasion capacity in skin SCC cell line. It is appealing to further design experiments in animals exposed to radiation to disclose the dose-effect relationship and/or the time course of the changes in the expression of RIPK4 related to proliferation and differentiation in skin SCC.

## Conclusions

Taken together, depletion of RIPK4 parallels higher malignancy potential in cutaneous SCC. RIPK4 is identified as an novel prognostic factor and a promising therapeutic target in skin SCC, which may be used to identify the risks of patients and guide individualized treatments. Further studies are needed regarding the mechanism by which RIPK4 integrates upstream signals to trigger specific responses to regulate epidermal tumorigenesis at the molecular level.

## Supplemental Information

10.7717/peerj.12932/supp-1 Supplemental Information 1 The expression level of RIPK4 after transfected with RIPK4 siRNA and negative control siRNA.

10.7717/peerj.12932/supp-2 Supplemental Information 2 The expression level of RIPK4 and β-actin by western blotting.

10.7717/peerj.12932/supp-3 Supplemental Information 3 Raw data for figure 2 and figure 3.

10.7717/peerj.12932/supp-4 Supplemental Information 4 The expression level of β-actin by western blotting.

10.7717/peerj.12932/supp-5 Supplemental Information 5 Raw data of western blotting.

## Additional Information and Declarations

### Data Availability

The following information was supplied regarding data availability: The raw data of the western blot is available in the Supplemental Files.
