# Supplementary figures and images for: Depletion of RIPK4 parallels higher malignancy potential in cutaneous squamous cell carcinoma
Source: PeerJ. 2022 Feb 10;10:e12932. doi: 10.7717/peerj.12932 (PMC8841032; doi:10.7717/peerj.12932)

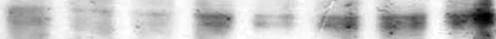

Supplement: Supplemental Information 1 [file peerj-10-12932-s001.jpg]

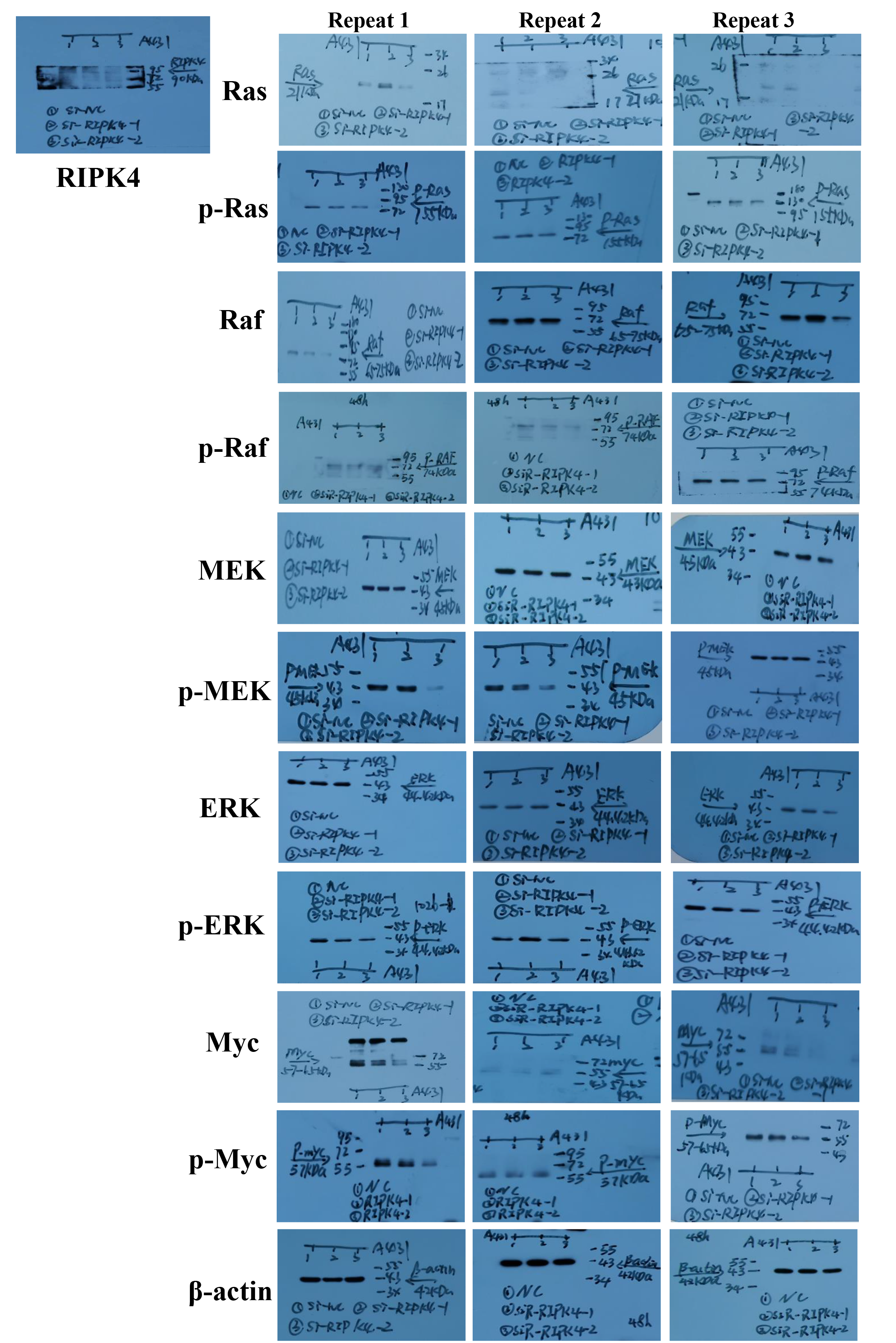

Supplement: Supplemental Information 2 [file peerj-10-12932-s002.png]

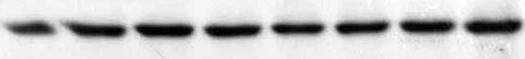

Supplement: Supplemental Information 4 [file peerj-10-12932-s004.jpg]

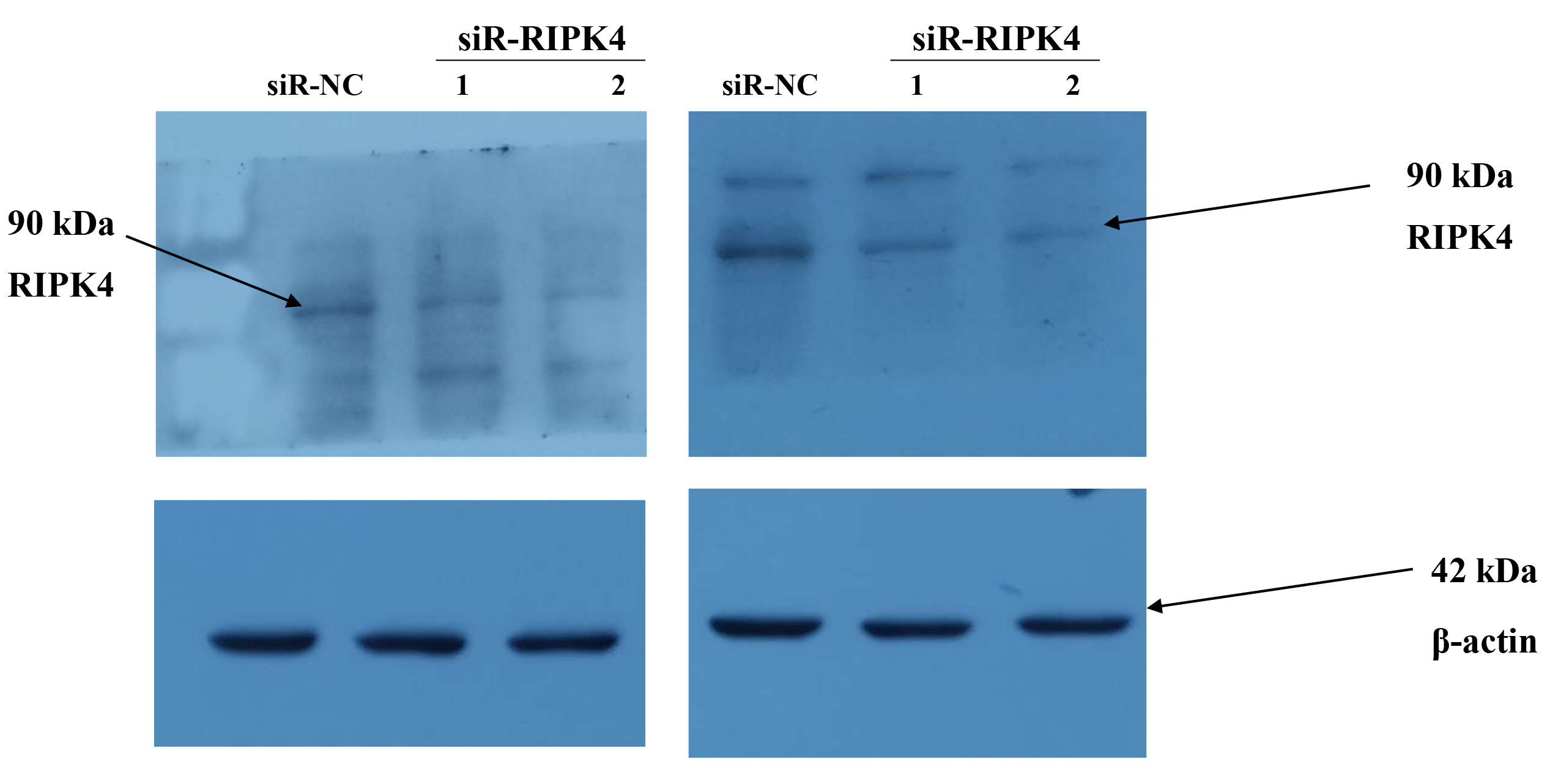

Supplement: Supplemental Information 5 [file peerj-10-12932-s005.png]
